# Real-world *EGFR* testing practices for non-small-cell lung cancer by thoracic pathology laboratories across Europe

**DOI:** 10.1016/j.esmoop.2023.101628

**Published:** 2023-09-14

**Authors:** P. Hofman, F. Calabrese, I. Kern, J. Adam, A. Alarcão, I. Alborelli, N.T. Anton, A. Arndt, A. Avdalyan, M. Barberis, H. Bégueret, B. Bisig, H. Blons, P. Boström, L. Brcic, G. Bubanovic, A. Buisson, A. Caliò, M. Cannone, L. Carvalho, C. Caumont, A. Cayre, L. Chalabreysse, M.P. Chenard, E. Conde, M.C. Copin, J.F. Côté, N. D’Haene, H.Y. Dai, L. de Leval, P. Delongova, M. Denčić-Fekete, A. Fabre, F. Ferenc, F. Forest, F. de Fraipont, M. Garcia-Martos, G. Gauchotte, R. Geraghty, E. Guerin, D. Guerrero, S. Hernandez, P. Hurník, B. Jean-Jacques, K. Kashofer, D. Kazdal, S. Lantuejoul, C. Leonce, A. Lupo, U. Malapelle, R. Matej, J.L. Merlin, K.D. Mertz, A. Morel, A. Mutka, N. Normanno, P. Ovidiu, A. Panizo, M.G. Papotti, E. Parobkova, G. Pasello, P. Pauwels, G. Pelosi, F. Penault-Llorca, T. Picot, N. Piton, A. Pittaro, G. Planchard, N. Poté, T. Radonic, I. Rapa, A. Rappa, C. Roma, M. Rot, J.C. Sabourin, I. Salmon, S. Savic Prince, A. Scarpa, E. Schuuring, I. Serre, V. Siozopoulou, D. Sizaret, S. Smojver-Ježek, J. Solassol, K. Steinestel, J. Stojšić, C. Syrykh, S. Timofeev, G. Troncone, A. Uguen, S. Valmary-Degano, A. Vigier, M. Volante, S.G.F. Wahl, A. Stenzinger, M. Ilié

**Affiliations:** 1 Laboratory of Clinical and Experimental Pathology, FHU OncoAge, Biobank Côte d’Azur BB-0033-00025, Louis Pasteur Hospital, IRCAN, Université Côte d'Azur, Nice, France; 2 Department of Cardiac, Thoracic, Vascular Sciences and Public Health, University of Padova, Padova, Italy; 3 Department of Pathology, University Clinic Golnik, Golnik, Slovenia; 4 Department of Pathology, Groupe Hospitalier Paris Saint-Joseph, Paris, France; 5 IAP-PM, Institute of Anatomical and Molecular Pathology, Faculty of Medicine, University of Coimbra, 3004-504 Coimbra, Portugal; 6 Department of Pathology, Institute of Medical Genetics and Pathology, University Hospital Basel, University of Basel, Basel, Switzerland; 7 Department of Genetics, University Hospital Bichat-Claude Bernard, Paris University, Paris, France; 8 Institute of Pathology and Molecular Pathology, Bundeswehrkrankenhaus Ulm, Oberer Eselsberg 40, 89081 Ulm, Germany; 9 Multidisciplinary Clinical Center “Kommunarka” of the Moscow Health Department, Moscow, Russia; 10 Oncogenomics Unit, European Institute of Oncology (IEO), Istituto di Ricovero e Cura a Carattere Scientifico (IRCCS), Milan, Italy; 11 Department of Pathology, University Hospital of Bordeaux, Hôpital Haut-Lévêque, Pessac, France; 12 Institute of Pathology, Department of Laboratory Medicine and Pathology, Lausanne University Hospital and University of Lausanne, Lausanne, Switzerland; 13 Pharmacogenomics and Molecular Oncology Unit, Biochemistry Department, Assistance Publique-Hopitaux de Paris, Hôpital Européen Georges Pompidou, Paris, France; 14 Department of Pathology, Turku University Hospital, Turku, Finland; 15 Diagnostic and Research Institute of Pathology, Medical University of Graz, Graz, Austria; 16 Laboratory for Molecular Pathology, Department of Pathology, University of Zagreb School of Medicine and University Hospital Centre Zagreb, Zagreb, Croatia; 17 Department of Biopathology, Centre Léon Bérard, Lyon, France; 18 Department of Diagnostics and Public Health, Section of Pathology, University and Hospital Trust of Verona, Verona, Italy; 19 Inter-Hospital Pathology Division, Istituto di Ricovero e Cura a Carattere Scientifico (IRCCS), MultiMedica, Milan, Italy; 20 Department of Tumor Biology, University Hospital of Bordeaux, Hospital Haut-Lévêque, Pessac, France; 21 Department of Biopathology, Jean Perrin Centre, Clermont-Ferrand, France; 22 Department of Pathology, Groupement Hospitalier Est, Bron, France; 23 Department of Pathology, University Hospital of Strasbourg, 67098 Strasbourg, France; 24 Department of Pathology, 12 de Octubre University Hospital, Universidad Complutense de Madrid, Research Institute 12 de Octubre University Hospital (i+12), CIBERONC, Madrid, Spain; 25 Department of Pathology, Université d'Angers, Centre Hospitalier Universitaire d’Angers, Angers, France; 26 Department of Pathology, Institut Mutualiste Montsouris, Paris, France; 27 Department of Pathology, Erasme Hospital, HUB ULB, Brussels, Belgium; 28 Department of Pathology, St. Olav’s Hospital, Trondheim University Hospital, Trondheim, Norway; 29 Institute of Molecular and Clinical Pathology and Medical Genetics, Faculty of Medicine, University Hospital Ostrava, Ostrava, Czech Republic; 30 Institute for Pathology Medical Faculty, Belgrade, Serbia; 31 Department of Histopathology, St. Vincent's University Hospital, University College Dublin School of Medicine, Dublin, Ireland; 32 Department of Pathology, University of Oradea, Oradea, Romania; 33 Department of Pathology, University Hospital of Saint-Etienne, Saint-Etienne, France; 34 Medical Unit of Molecular Genetic (Hereditary Diseases and Oncology), Grenoble University Hospital, Grenoble, France; 35 Department of Pathology, Gregorio Marañón General University Hospital, Madrid, Spain; 36 Department of Biopathology, CHRU-ICL, CHRU Nancy, Vandoeuvre-lès-Nancy, France; 37 Department of Molecular Cancer Genetics, Laboratory of Biochemistry and Molecular Biology, University Hospital of Strasbourg, Strasbourg, France; 38 Biomedical Research Centre, Navarra Health Service, Pamplona, Navarra, Spain; 39 Department of Pathology, CHU de Caen Côte de Nacre, Caen, France; 40 Institute of Pathology, Heidelberg University Hospital, Heidelberg, Germany; Translational Lung Research Center Heidelberg (TLRC-H), member of the German Center for Lung Research (DZL), Heidelberg, Germany; 41 Department of Biopathology, Centre Leon Berard Unicancer and Pathology Research Platform, Cancer Research Center of Lyon (CRCL), Lyon, France; 42 Department of Pathology, Hôpital Cochin, Assistance Publique-Hôpitaux de Paris, Université de Paris, Paris, France; 43 Department of Public Health, University of Naples Federico II, Naples, Italy; 44 Department of Pathology and Molecular Medicine, Thomayer University Hospital, Prague, Czech Republic; 45 Department of Biopathology, Institut de Cancérologie de Lorraine, University of Lorraine, Vandoeuvre-Les-Nancy, France; 46 Institute of Pathology, Cantonal Hospital Baselland, Liestal, Switzerland; 47 Department of Innate Immunity and Immunotherapy, Institut de Cancérologie de l'Ouest - Centre Paul Papin, Angers, France; 48 HUSLAB, Department of Pathology, Helsinki University Hospital, Helsinki, Finland; 49 Cell Biology and Biotherapy Unit, INT-Fondazione Pascale, Via M. Semmola, Naples, Italy; 50 Department of Pathology, Complejo Hospitalario de Navarra, Pamplona, Navarra, Spain; 51 Division of Pathology, University Hospital Città Della Salute, Turin, Italy; 52 Division of Medical Oncology 2, Veneto Institute of Oncology IOV-IRCCS, Padova, Italy; 53 Department of Pathology, University Hospital Antwerp and University of Antwerp, Antwerp, Belgium; 54 Department of Oncology and Hemato-Oncology, University of Milan, Milan, Italy; 55 Department of Pathology, Clermont Auvergne University, “Molecular Imaging and Theranostic Strategies”, Center Jean Perrin, Montalembert, Clermont-Ferrand, France; 56 Department of Pathology, Rouen University Hospital, France and Normandie University, UNIROUEN, Inserm U1245, Rouen, France; 57 Department of Pathology, Hospital Bichat Bichat, Assistance Publique Hôpitaux de Paris; Université Paris Cité, Paris, France; 58 Department of Pathology, Amsterdam University Medical Center, VUMC, University of Amsterdam, Amsterdam, Netherlands; 59 Pathology Unit, San Luigi Hospital, Orbassano Turin, Italy; 60 Department of Pathology, Erasme Hospital, HUB ULB, Brussels, Belgium; CurePath, Jumet, Belgium; 61 Department of Pathology, University Medical Center Groningen, University of Groningen, Groningen, Netherlands; 62 Department of Pathology, Gui de Chauliac Hospital, Montpellier University Medical Center, University of Montpellier, 80 Avenue Augustin Fliche, Montpellier, France; 63 Department of Pathology, CHRU Tours - Hôpital Trousseau, Chambray-lès-Tours, France; 64 Division for Pulmonary Cytology, Department of Pathology and Cytology, University Hospital Centre Zagreb, School of Medicine, University of Zagreb, Zagreb, Croatia; 65 Solid Tumour Laboratory, Pathology and Oncobiology Department, CHU Montpellier, University of Montpellier, Montpellier, France; 66 Department of Thoracic Pathology, Section of Pathology, University Clinical Centre of Serbia, Belgrade, Serbia; 67 Department of Pathology, IUC-T-Oncopole, Toulouse, France; 68 Department of Pathological Anatomy and Cytology, CHRU de Brest, Brest, France; LBAI, UMR1227, INSERM, University of Brest, CHU de Brest, Brest, France; 69 Department of Pathology, Institute for Advanced Biosciences, CHU Grenoble Alpes, Université Grenoble Alpes, Grenoble, France; 70 Department of Oncology, University of Turin, San Luigi Hospital, Orbassano, Turin, Italy

**Keywords:** EGFR, survey, Europe, molecular pathology, non-small-cell lung cancer

## Abstract

**Background:**

Testing for epidermal growth factor receptor (*EGFR*) mutations is an essential recommendation in guidelines for metastatic non-squamous non-small-cell lung cancer, and is considered mandatory in European countries. However, in practice, challenges are often faced when carrying out routine biomarker testing, including access to testing, inadequate tissue samples and long turnaround times (TATs).

**Materials and methods:**

To evaluate the real-world *EGFR* testing practices of European pathology laboratories, an online survey was set up and validated by the Pulmonary Pathology Working Group of the European Society of Pathology and distributed to 64 expert testing laboratories. The retrospective survey focussed on laboratory organisation and daily *EGFR* testing practice of pathologists and molecular biologists between 2018 and 2021.

**Results:**

TATs varied greatly both between and within countries. These discrepancies may be partly due to reflex testing practices, as 20.8% of laboratories carried out *EGFR* testing only at the request of the clinician. Many laboratories across Europe still favour single-test sequencing as a primary method of *EGFR* mutation identification; 32.7% indicated that they only used targeted techniques and 45.1% used single-gene testing followed by next-generation sequencing (NGS), depending on the case. Reported testing rates were consistent over time with no significant decrease in the number of *EGFR* tests carried out in 2020, despite the increased pressure faced by testing facilities during the COVID-19 pandemic. ISO 15189 accreditation was reported by 42.0% of molecular biology laboratories for single-test sequencing, and by 42.3% for NGS. 92.5% of laboratories indicated they regularly participate in an external quality assessment scheme.

**Conclusions:**

These results highlight the strong heterogeneity of *EGFR* testing that still occurs within thoracic pathology and molecular biology laboratories across Europe. Even among expert testing facilities there is variability in testing capabilities, TAT, reflex testing practice and laboratory accreditation, stressing the need to harmonise reimbursement technologies and decision-making algorithms in Europe.

## Highlights

• Substantial heterogeneity exists in several aspects of EGFR mutation testing, including among expert testing facilities.
• Targeted sequencing is commonly used in conjunction with or in place of NGS.
• While the average TAT reported for EGFR mutation testing was 7-10 days, few laboratories reported TATs of up to 30 days.

## Introduction

Non-small-cell lung cancer (NSCLC) remains the most prevalent form of lung cancer, accounting for approximately 80%-90% of cases.^1^ The selection of optimal specific treatments for patients diagnosed with NSCLC has become increasingly complex as more treatment options are developed and shown to be effective for specific disease indications.^2^, ^3^, ^4^, ^5^ For example, for patients with a sensitising (L858R/exon 19 deletion, with or without a concomitant T790M mutation) epidermal growth factor receptor (*EGFR*) mutation with stage IV non-squamous NSCLC and a performance status score of 0-2 who have not had previous systemic therapy, a third-generation tyrosine kinase inhibitor (TKI) is the optimal first-line treatment.^2^ Therefore, it is no longer sufficient to rely on morphological diagnosis alone when determining the most appropriate treatment options.^6^

Molecular testing guidelines and recommendations in thoracic oncology are constantly shifting and have been updating rapidly in recent years. There has been increased emphasis on incorporating biomarker testing, and many guidelines now recommend testing for targetable mutations to select the optimal treatment option. Both the European Society for Medical Oncology (ESMO) and American Society of Clinical Oncology (ASCO) guidelines recommend that *EGFR* testing is essential in patients with metastatic non-squamous NSCLC.^2^^,^^6^^,^^7^ Moreover, third-generation EGFR TKIs are approved for the adjuvant treatment of patients with completely resected, stage IB-IIIA, *EGFR* sensitising mutation-positive NSCLC.^8^

Although testing for *EGFR* mutations is now considered mandatory in European countries^9^^,^^10^ and is mandated in early-stage disease (by ESMO),^11^ in practice, considerable challenges are often faced when carrying out routine biomarker testing, including inadequate tissue samples, long turnaround times (TATs), lack of access to testing [notably to next-generation sequencing (NGS) testing], lack of implementation of additional testing techniques such as liquid biopsy, and inconsistent reimbursement of diagnostic tests between different European countries. Pathology laboratories, particularly those dealing with respiratory tract specimens, have also faced considerable challenges in recent years with increased testing demand and the impact of the coronavirus disease 2019 (COVID-19) pandemic.^12^ Given these pressures, it is important to fully understand how pathology laboratories are implementing recommended testing for *EGFR* in different stages of NSCLC, and how they are dealing with the potential increase in demand for testing.^13^

This study aimed to evaluate the real-world daily practices of thoracic pathology laboratories across Europe concerning *EGFR* testing, with attention given to techniques used, testing TATs, and changes to treatment and testing rates. The broad range of the survey results promotes advocating for harmonisation in practices and provides a basis for discussion to establish European guidelines in this field.

## Materials and methods

An online survey (https://fr.surveymonkey.com/) was sent to 64 expert testing laboratories across Europe (Supplementary Figure S1, available at https://doi.org/10.1016/j.esmoop.2023.101628). The survey was developed and validated by the Pulmonary Pathology Working Group of the European Society of Pathology together with ESMO representatives. All thoracic pathologists from the laboratories approached are members of the Pulmonary Pathology Working Group of the European Society of Pathology. This retrospective survey focussed on the laboratory organisation and daily practice of pathologists and molecular biologists between 2018 and 2021 to gain insights into the real-world *EGFR* testing practices of pathology laboratories across Europe.

The survey was divided into sections to incorporate questions covering (i) the clinical circumstances in which *EGFR* testing is carried out and the types of samples, (ii) molecular biology techniques carried out, (iii) percentage of tumour cells on the samples required for analysis, (iv) average TAT for *EGFR* mutation status, (v) annual rates of *EGFR* testing and aggregated results, (vi) laboratory accreditation/certification, (vii) external quality assessment (EQA), and (viii) treatment directions for patients, if known (Supplementary Table S1, available at https://doi.org/10.1016/j.esmoop.2023.101628).

Reflex testing is defined as the process in which the pathologist orders a group of preapproved biomarkers (e.g. *EGFR* gene mutations) for genetic profiling at the time of initial diagnosis, without referral back to the oncologist.

### Statistical analysis

Numerical variables are expressed as mean (standard deviation) or median [interquartile range (IQR)] and compared with either Welch's *t*-test (or its non-parametric alternative Wilcoxon rank-sum test) or ANOVA (analysis of variance; or its non-parametric alternative Kruskal–Wallis test) where appropriate. Categorical variables are expressed as *n* (%) and compared with the chi-square test or its non-parametric alternative Fisher's test with simulated *P* values. Statistical tests and representations of the data were carried out using the StatAid software. *P* values are not adjusted.

## Results

The survey was returned by 53 of the 64 (82.8%) pathology laboratories invited to participate. These laboratories are considered expert testing laboratories in thoracic pathology in Europe in their respective countries. Laboratories from a total of 17 European countries participated in the survey and participants per country ranged from 1 to 22 (Supplementary Figure S1, available at https://doi.org/10.1016/j.esmoop.2023.101628). It should be noted that not all laboratories were able to provide data for every question; therefore, the *n* number differs and is reported in each instance.

### *Clinical situations of* EGFR *testing and types of samples analysed*

**Figure 1:**
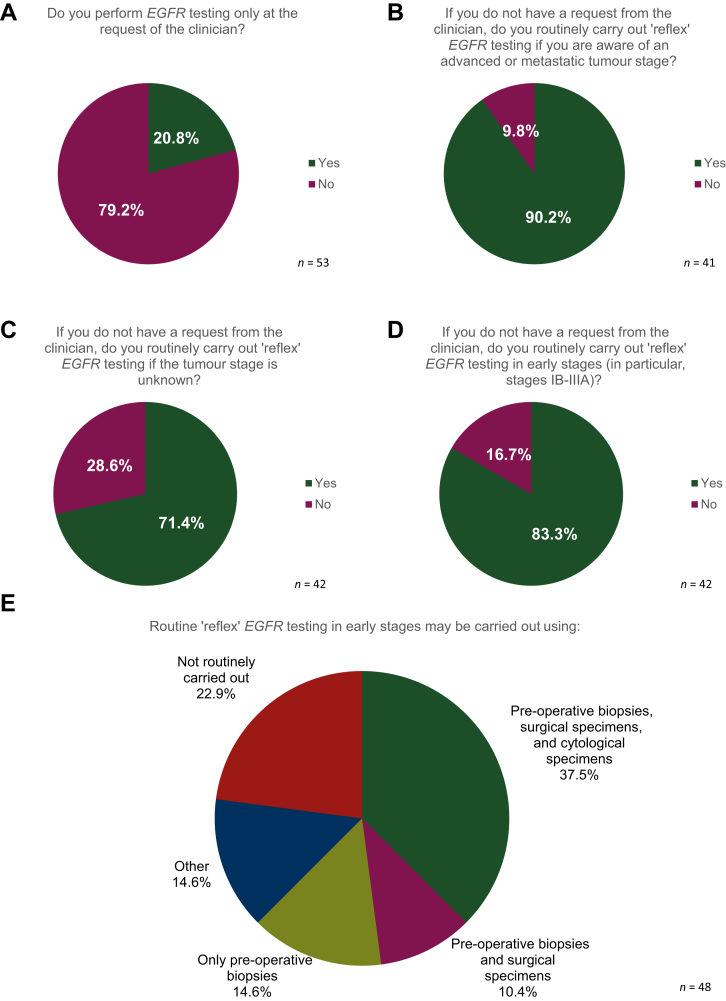
Results of the survey related to reflex *EGFR* testing according to stage. EGFR, epidermal growth factor receptor gene.

Independently of the tumour–node–metastasis (TNM) stage, the majority of laboratories (79.2%, 42/53) indicated carrying out ‘reflex’ *EGFR* testing (Figure 1). Of these laboratories, reflex testing was carried out by 90.2% (37 of the 41 laboratories that submitted a response to this question) if they were aware of advanced or metastatic tumour stage, by 71.4% (30/42) even if tumour stage was unknown and by 83.3% (35/42) not only in advanced but also in early-stage disease (particularly stage IB-IIIA) (Figure 1). For laboratories carrying out reflex testing in early stages, the median start date for this testing was January 2020 (range from April 2007 to January 2022).

More than one-third of the laboratories (18/48, 37.5%) indicated that they carried out reflex *EGFR* testing in early stages on pre-operative biopsies, surgical specimens, and/or cytological specimens.

The majority [62.2% (33/53)] of participating laboratories indicated that they routinely carry out reflex *EGFR* testing in non-squamous NSCLC, while 28.3% (15/53) carried out reflex testing in adenocarcinoma and 13.2% (7/53) in all histological subtypes, including squamous cell carcinoma (data not shown). Reflex testing decisions for histological subtype may also be influenced by current approved indications of available therapeutic options, as the use of osimertinib is restricted to adjuvant therapy following complete tumour resection in adult patients with stage IB-IIIA (TNM staging system for lung cancer seventh edition as per the ADAURA clinical trial) non-squamous cell lung carcinoma, harbouring *EGFR* exon 19 deletions or exon 21 (L858R) substitution mutations.^7^^,^^8^

In addition, 46.1% (24/52) of the laboratories routinely carry out *EGFR* testing on liquid biopsies at both diagnosis and at tumour progression in advanced or metastatic NSCLC, and 25.0% (13/52) at tumour progression only, whereas 28.9% (15/52) of the laboratories do not carry out *EGFR* testing on liquid biopsies from patients with NSCLC (data not shown). In most cases, liquid biopsy replaced tissue analysis in certain clinical situations (insufficient amount of tumour cells typically < 5%, exhausted biopsy, failure to carry out a tissue biopsy).

**Figure 2:**
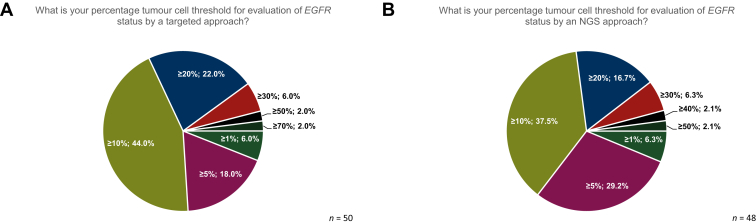
**Percentage tumor cell thresholds.** Different thresholds were used in the laboratories for the evaluation of *EGFR* status by (A) both single-test sequencing, and (B) NGS approaches. EGFR, epidermal growth factor receptor gene; NGS, next-generation sequencing.

A tumour cell threshold of ≥10% is most commonly used among the laboratories (22/50, 44.0%) for evaluating the *EGFR* status with a single-gene testing approach. An additional 22.0% (11/50) of the laboratories indicated that they use a tumour cell threshold of ≥20%, and 18.0% (9/50) used a tumour cell threshold of ≥5% (Figure 2A). A minority of laboratories used a higher threshold for evaluating *EGFR* status with a single-test approach (3/50 used ≥30%, 1/50 used ≥50% and 1/50 used ≥70%), while 6.0% (3/50) used a tumour cell threshold of ≥1% (Figure 2A).

When using an NGS approach to evaluate *EGFR* status, 37.5% (18/48) of laboratories used a tumour cell threshold of ≥10%, 29.7% (14/48) used ≥5%, and 16.7% (8/48) used a threshold of ≥20% (Figure 2B).

### Molecular biology techniques carried out

**Figure 3:**
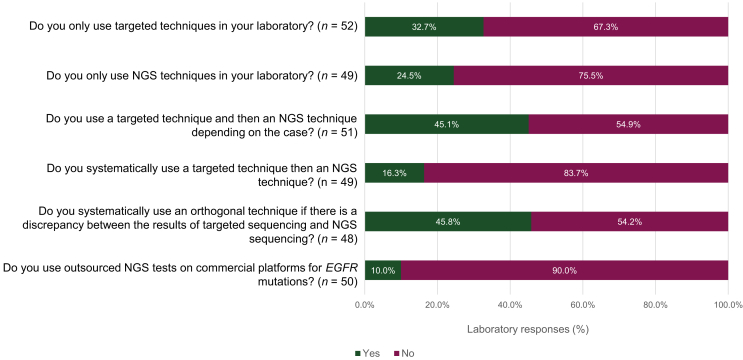
Overview of the molecular methodologies used in the surveyed laboratories. EGFR, epidermal growth factor receptor gene; NGS, next-generation sequencing.

The use of a single-test sequencing technique followed by NGS (depending on the case) was used by 45.1% of participating laboratories (23/51) (Supplementary Table S2, available at https://doi.org/10.1016/j.esmoop.2023.101628). In the case of a discrepancy between the results of single-test sequencing and NGS, 45.8% of laboratories (22/48) indicated that they would systematically use an orthogonal technique. A total of 32.7% of laboratories (17/52) responded that they only used single-test sequencing techniques, and 24.5% (12/49) only used NGS techniques (Figure 3).

Laboratories reporting the use of single-gene testing for tissue or cytological samples (84.9%, 45/53) used a variety of techniques, with some indicating the use of more than one technique in-house. The most commonly used techniques were real-time polymerase chain reaction (RT-PCR), commercialised from Biocartis (Idylla™ kits, Biocartis, Mechelen, Belgium) (36.4%, 20 of the 55 total responses), and cobas® (Roche, Basel, Switzerland) (20.0%, 11/55) or droplet digital polymerase chain reaction (ddPCR) (7.3%, 4/55). In addition, single-test sequencing was used by 66.0% (35/53) of the laboratories to test for *EGFR* mutations using liquid biopsies, with a large variety of techniques used. The most commonly used techniques for liquid biopsy testing were cobas® (35.9%, 14 of the 39 total responses), ddPCR (17.9%, 7/39), and NGS (15.4%, 6/39).

In total, 81.1% (43/53) of surveyed laboratories used NGS techniques to search for *EGFR* mutations between 2018 and 2021, with the majority (76.6%, 36/47) using amplicon-based assays and 21.3% (11/47) using hybrid capture–based methods. Of those laboratories, 65.9% (29/44) used NGS on tissue, cytology, and blood samples; 29.5% (13/44) used NGS on tissue only, and 4.5% (2/44) on tissue and blood.

### Turnaround time for *EGFR* status

**Figure 4:**
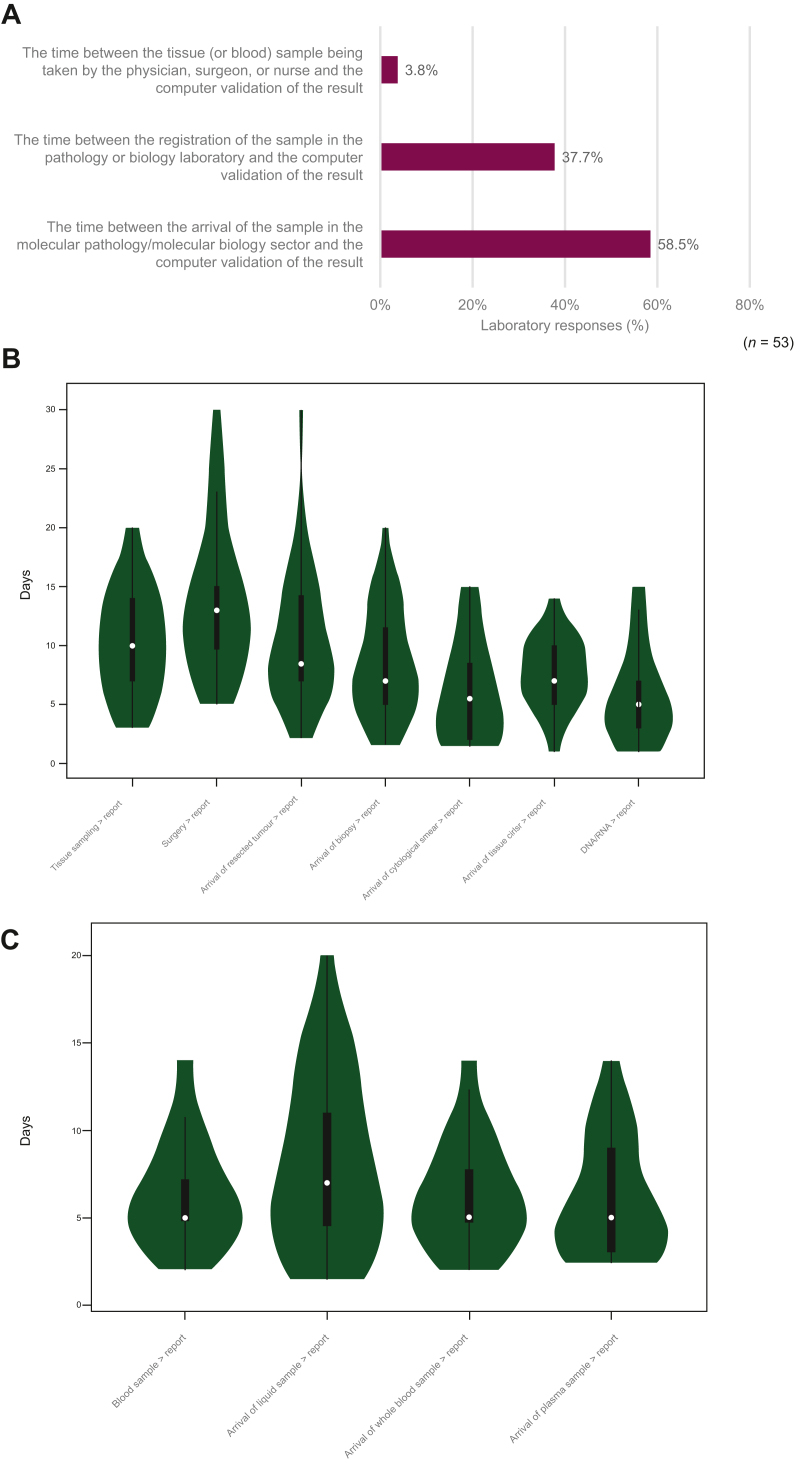
**Survey on the definition of the turnaround time.** The definition was considered in different scenarios for (A) an *EGFR* mutation analysis, (B) obtaining *EGFR* testing results from tissue samples, and (C) obtaining *EGFR* testing results from liquid biopsy samples. Violin plots illustrates Kernal density (green), data range (thin line), IQR (bold line), and mean (white). EGFR, epidermal growth factor receptor gene; IQR, interquartile range.

Participants were surveyed on how they would define the time to results in their institution, with the majority (58.5%, 31/53) responding that they considered the definition to be the time between a sample arriving at the molecular pathology sector and the validation of the molecular pathology report. Alternatively, 37.7% (20/53) of laboratories defined the time to results as the time between registration of the sample in the pathology or biology laboratory and the validation of the molecular pathology report, and 3.8% (2/53) defined it as the time between the tissue or blood sample collection in the clinical or surgical department and the validation of the molecular pathology report (Figure 4A).

A large variability in the estimated average TATs was reported both between countries and across laboratories within the same country. On average, *EGFR* testing using liquid biopsy samples resulted in a faster (*P* < 0.001) time to results (mean 6.7 days; median 5 days) when compared with testing using tissue or cytology samples (mean 9.0 days; median 8 days) (Figure 4B and C). Although some laboratories reported TATs of up to 30 days, the average TAT for surveyed laboratories was between 7 and 10 days, depending on the testing scenario. Although the suggested TAT in the clinical guidelines for molecular testing is 10 working days (between sample receipt and reporting of all results),^14^ it is worth noting that only expert laboratories were invited to participate in this study, which could explain some of the observed differences.

### Annual rates of *EGFR* testing and testing results

**Figure 5:**
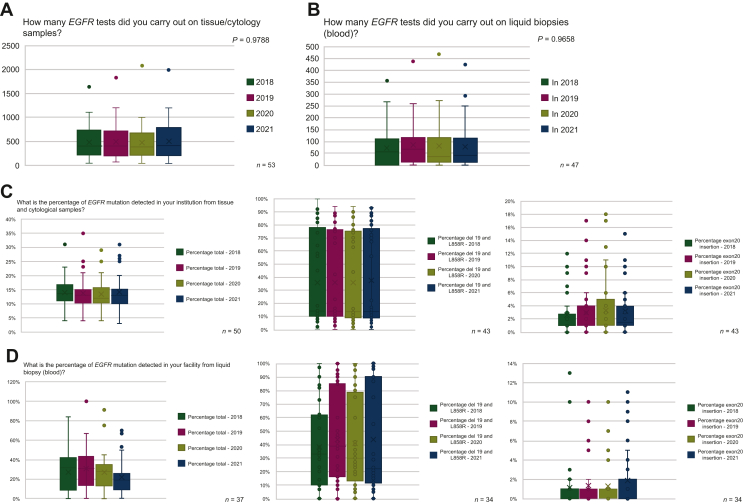
**Overview of annual testing rates for EGFR in tissue and liquid biopsy samples.** Histograms illustrate mean (X), IQR (horizontal lines in bars), data range (error bars), and data outliers (dots). EGFR, epidermal growth factor receptor gene; IQR, interquartile range.

It was hypothesised that fluctuations in testing rates for tissue, cytology, and blood samples may be observed in 2020, given the significant disruption and increased pressure faced by molecular biology and pathology laboratories across Europe due to the COVID-19 pandemic.^11^ There was a slight but non-significant decrease in *EGFR* testing rates reported for tissue/cytology samples in 2020, with a gradual resumption in testing rate seen in 2021 (Figure 5A). A potential influence of the US Food and Drug Administration (FDA) and/or the European Medicines Agency (EMA) recommendations for *EGFR* tissue testing in early-stage disease was also observed in the tissue testing rates reported in 2021. There was, however, no observable impact on blood sample testing rates in 2020, and no impact of the US FDA/EMA recommendations for testing in early stages was seen for liquid biopsy in 2021 (Figure 5B).

There were no significant differences in the total reported percentages of *EGFR* mutations detected by laboratories between 2018 and 2021, in either tissue or cytological samples (Figure 5C), or liquid biopsy (Figure 5D). There were similarly no significant differences in the percentage of del19 and L858R, or exon 20 insertions detected in either tissue/cytology or blood samples between 2018 and 2021.

### Laboratory accreditation

Given that the laboratories participating in this survey are considered expert testing laboratories in Europe, the number with International Organization for Standardization (ISO) 15189 accreditation might be considered relatively low, with 42.0% (21/50) of molecular biology laboratories ISO 15189 accredited for single-gene testing, 42.3% (30/52) ISO 15189 accredited for NGS, and 38.5% (20/52) of surgical pathology laboratories ISO 15189 accredited (Supplementary Figure S2, available at https://doi.org/10.1016/j.esmoop.2023.101628). However, although a number of laboratories indicated that their surgical pathology and molecular biology laboratories were not accredited according to ISO 15189, some respondents stated that their laboratories were accredited to other standards (e.g. ISO 17020).

### External quality assessments

In total, 92.5% (49/53) of laboratories indicated that they have participated in at least one EQA scheme, and of the laboratories that participated, 93.9% (46/49) took part annually. The majority of laboratories (86.1%, 31/36) indicated that they first started participating in an EQA scheme in 2017 or earlier. The majority of laboratories participated in EQAs for NGS (71.7%, 38/53), and 47.2% (25/53) also participated in an EQA scheme for quantitative PCR (Supplementary Table S3, available at https://doi.org/10.1016/j.esmoop.2023.101628).

### Treatment

Laboratories were also surveyed on the number of patients found to have an *EGFR* mutation who went on to receive a targeted treatment with an EGFR inhibitor at their institution, but these data were often reported to be unavailable or unknown to clinical and molecular laboratory staff (60.4%, 32/53 of respondents were unable to provide data). Where values could be provided, the majority of laboratories reported that consistent proportions of patients were treated each year in their institutions between 2018 and 2021.

Laboratories reported that targeted therapy with an EGFR TKI was only offered to patients with an *EGFR* mutation in the adjuvant setting from 2021. No significant trends in the number of institutions offering second-line or first-line EGFR TKIs were observed between 2018 and 2021. There was an observable increase in the number of patients receiving third-generation EGFR TKIs from 2020, with corresponding decreases in the number of patients offered first- and second-generation EGFR TKIs after 2020.

## Discussion

This retrospective study highlights the heterogeneity of *EGFR* testing that exists within thoracic pathology and molecular pathology laboratories across Europe. Even among expert testing facilities, there remains large variability in available testing modalities, TAT, and laboratory accreditation.

Current recommendations for *EGFR* testing highlight the need for an appropriate, validated method.^10^^,^^12^^,^^13^ Despite current international guidelines, strong evidence, and cost-effectiveness of upfront NGS testing,^2^^,^^3^^,^^5^^,^^6^^,^^8^, ^9^, ^10^, ^11^^,^^15^ it was found that *EGFR* assessment in Europe is carried out through a large variety of *EGFR* mutation detection techniques, with many laboratories across Europe still favouring single-gene testing as a primary method of *EGFR* mutation identification. Nearly a third (32.7%) of surveyed laboratories indicated that they only used single-gene testing, and a further 45.1% indicated that they used single-gene test followed by NGS, depending on the case. NGS testing was not available in all laboratories, and where NGS testing was possible the cost of running the tests or the increased TAT may be a prohibitive factor. Thus, there remains significant discrepancies in access to or use of NGS across Europe, where reimbursement constraints and limited resources in academic laboratories are key limitations for adoption of best practice in *EGFR* testing.^16^^,^^17^

In Europe, it is not common practice for laboratories to send out testing to centralised molecular facilities. In fact, only a small percentage of laboratories, ∼10%, reported that they outsource *EGFR* testing to commercial providers. This suggests that most expert laboratories in Europe conduct *EGFR* testing in-house. While outsourcing may have benefits such as cost savings and access to specialised equipment, it appears that many laboratories prefer to maintain control over their testing processes by conducting them in-house. It is important for laboratories to carefully evaluate the costs and benefits of outsourcing, considering factors such as TAT, quality control, and the potential impact on patient care. Ultimately, the decision of whether to outsource testing or keep it in-house will depend on the specific needs and resources of each laboratory, complexity of tests, innovation power of the laboratory, sufficient expertise to carry out testing, e.g. needed for more complex assays regarding also variant calling, interpretation, and bioinformatics.^18^

Guideline-recommended molecular testing in NSCLC involves not only testing for EGFR; multigene testing is becoming quite common (and probably a requirement; see remarks on costs). Taking this into account, consideration should be given to including *EGFR* in NGS panels.

This study identified a number of bottlenecks, with a large range of estimated average TATs between arrival of a sample at the pathology department and validation of the molecular pathology report. However, the definition of TAT seems not to be fully grasped and needs more clarity in international guidelines. Moreover, the TAT for getting the molecular results varies depending on different parameters (notably the type of methods, reflex or bespoke testing, etc.). The estimated TATs varied both between and within countries, and time to results was slightly but not significantly faster for liquid biopsy samples (mean of 7 days, IQR 4.5-12.5 days) when compared with tissue samples (mean of 7 days, IQR 5-15 days) but were reported to take as long as 20 or 30 working days, respectively. Discrepancy in TATs between institutions may be partly due to reflex testing practices, as 20.8% (11/53) of laboratories reported carrying out *EGFR* testing only at the request of the clinician.

Liquid and tissue biopsies show similar TAT, contrary to expectations that liquid biopsy would be faster. The processing, analysis, and interpretation of liquid biopsy samples involve multiple steps and techniques, contributing to the overall TAT. Tissue biopsy platforms have improved, and technologies like NGS have sped up analysis. Liquid biopsy interpretation is complex due to limited genetic material. TAT can vary based on facility, protocols, and workload. Ongoing advancements may enhance liquid biopsy efficiency in the future. The choice of tumour cell threshold depends on the assay being used. In single-test sequencing, 66% of laboratories use a threshold of ≥10% or ≥20%, while for NGS approaches, only 55% use these thresholds. Moreover, a relatively high percentage of laboratories use a less stringent tumour cell threshold of ≥5% (18% for single-test sequencing and 29% for NGS). However, this may lead to cytosine deamination artefacts in the context of formalin-fixed, paraffin-embedded (FFPE) samples.^19^^,^^20^ Therefore, laboratories should carefully consider the appropriate threshold for their specific assay and the potential impact on accuracy and reliability of results, particularly when dealing with FFPE samples.

A significant proportion of laboratories (79%) reported conducting reflex *EGFR* testing, which increased to 90% when laboratories were aware of advanced or metastatic tumour stage. This highlights the importance of effective communication between molecular pathologists and referring physicians,^21^ to ensure that the appropriate testing is conducted and that results are accurately interpreted in the context of the patient's condition. It is crucial for molecular pathologists and referring physicians to work together closely to provide optimal care for patients. Furthermore, the study also found that a large majority of the participating laboratories adhere to international guidelines and the latest advances in thoracic oncology. They routinely carry out reflex *EGFR* testing in non-squamous NSCLC cases.^7^^,^^8^ This commitment to staying up to date with advancements in the field emphasises the importance of incorporating the latest evidence-based practices into patient care.

Testing rates remained reasonably consistent over time, with only a slight, non-significant decrease in the number of *EGFR* tests carried out in 2020, despite the increased pressure and difficulties faced by thoracic pathology testing facilities during the COVID-19 pandemic. Testing rates recovered to pre-pandemic levels by 2021, and this may be due, in part, to the introduction by the US FDA/EMA of a recommendation for *EGFR* testing to be carried out in early-stage NSCLC.

The survey identified a number of weaknesses in current *EGFR* testing practices in Europe. Although most laboratories reported participation in an EQA scheme, around 8% of laboratories (4/53) did not. Such schemes are essential for ensuring consistently high testing standards and their importance is recognised in testing guidelines and consensus documents.^13^^,^^22^ A large proportion of laboratories also reported that they did not have ISO 15189 accreditation, for either molecular pathology or surgical pathology laboratories. There were also discrepancies in the laboratory accreditation attained by different testing facilities both between and within countries across Europe. Although some laboratories indicated that they were accredited to other ISO standards (e.g. ISO 17020), homogeneity in the standards followed by laboratories would help to ensure consistent testing quality and competence. EQA programmes have illustrated that accreditation status is also associated with successful participation in *EGFR* mutation analysis.^23^

This was a retrospective study, investigating *EGFR* testing practices of laboratories across Europe between 2018 and 2021; as such, some limitations of this study should be recognised. In particular, it should be noted that the treatment outcomes for patients were often not known to molecular biologists and pathologists, highlighting the need for improved communication between departments within facilities and to build central database collecting of molecular results. Most laboratories were not able to answer all questions, leaving questions around ease of access to data/communication between departments in general. There is also substantial heterogeneity in the organisation of testing facilities; in some facilities, pathologists or molecular biologists may work closely, facilitating effective communication between faculty members, but in other institutions, these laboratories may be located separately, with potentially adverse consequences for communication and sample handling times. Only expert testing laboratories were selected to take part in this study, and there may be even greater variability in standard practice and access to testing in smaller facilities and community hospitals. Laboratories were also only given the option to specify if they had achieved ISO 15189 accreditation; however, some laboratories indicated that they are accredited to other accreditation norms, which were not included within the survey but may warrant consideration. The transferability of the survey results to all European countries is limited due to the predominant representation of French and Italian participating centres. It is important to consider this as a significant limitation.

The results of this survey highlight the need for increased communication between clinicians and pathologists or molecular biologists working in the same institutions and within the same regions, and it is believed that a number of improvements in testing practices could be implemented. *EGFR* testing in NSCLC is known to be essential for identifying targetable mutations and ensuring all patients are receiving the most appropriate treatment for their cancer type. Greater involvement of patients, advocacy groups, and stakeholders may be necessary to drive the changes required for improvements in access to *EGFR* testing and ensure that all patients are receiving the same high-quality care. Although it is important to harmonise *EGFR* testing practices across facilities to avoid discrepancies in patient access to testing, it is also recognised that cost and reimbursement considerations differ between countries, and this has a significant impact on *EGFR* testing algorithms.^24^

Future testing frequency will depend on treatment regimen, disease progression, and patient characteristics. Initial and recurrent EGFR testing is standard, but additional testing during treatment may be needed to identify emerging alterations. Various techniques like NGS, digital PCR, and allele-specific PCR can detect resistance mechanisms. Repeated testing has economic implications, but benefits in treatment optimisation and patient outcomes outweigh costs. Longitudinal molecular testing is crucial for EGFR-mutated lung cancer, and the optimal technique may involve NGS or targeted approaches. Despite European and international guidelines, some variability in laboratory practices exists, even among expert laboratories. This highlights the need to harmonise budgets and reimbursement across Europe, in addition to standardising technologies and decision-making algorithms. An increase in the practice of liquid biopsy testing for *EGFR* mutation detection both at diagnosis and at tumour progression may be beneficial for improved TATs.^25^ There is also an urgent need to increase the prevalence of NGS testing comparatively to single-test sequencing, considering the recommendations included in the ESMO guidelines for both tissue and cell-free DNA, and the increased risk of obtaining false-negative results when using single-test sequencing for *EGFR* genomic alteration detection. Moreover, using NGS enables identification of multiple other biomarkers relevant for the patients’ treatment using one test. Implementation of reflex testing protocols for NGS will improve the associated TATs for obtaining results and new NGS methodologies can further decrease this TAT.^26^^,^^27^ *EGFR* testing in NSCLC is no longer a stand-alone test, and we are moving towards multigene resting (e.g. most guidelines already recommend >10 targets in metastatic NSCLC). Thus, predictive testing will need to move into the direction of parallel NGS using large panels.
