# Supplementary material for: Real-world EGFR testing practices for non-small-cell lung cancer by thoracic pathology laboratories across Europe
Source: ESMO Open. 2023 Sep 14;8(5):101628. doi: 10.1016/j.esmoop.2023.101628 (PMC10594022; doi:10.1016/j.esmoop.2023.101628)
Supplement: Supplementary data [file mmc1.docx]

**SUPPLEMENTARY MATERIAL**

**Real-world *EGFR* testing practices for non-small cell lung cancer by thoracic pathology laboratories across Europe**

P. Hofman, F. Calabrese, I. Kern, J. Adam, A. Alarcão, I. Alborelli, N.T. Anton, A. Arndt, 
A. Avdalyan, M. Barberis, H. Bégueret, B. Bisig, H. Blons, P. Boström, L. Brcic, G. Bubanovic, A. Buisson, A. Caliò, M. Cannone, L. Carvalho, C. Caumont, A. Cayre, L. Chalabreysse, M.P. Chenard, E. Conde, M.C. Copin, J.F. Côté, N. D’Haene, H.Y. Dai, L. de Leval, P. Delongova, M. Denčić-Fekete, A. Fabre, F. Ferenc, F. Forest, F. de Fraipont, M. Garcia-Martos, G. Gauchotte, R. Geraghty, E. Guerin, D. Guerrero, S. Hernandez, P. Hurník, B. Jean-Jacques, K. Kashofer, D. Kazdal, S. Lantuejoul, C. Leonce, A. Lupo, U. Malapelle, R. Matej, J.L. Merlin, K.D. Mertz, A. Morel, A. Mutka, N. Normanno, P. Ovidiu, A. Panizo, M.G. Papotti, E. Parobkova, G. Pasello, P. Pauwels, G. Pelosi, F. Penault-Llorca, T. Picot, N. Piton, A. Pittaro, G. Planchard, N. Poté, T. Radonic, I. Rapa, A. Rappa, C. Roma, M. Rot, J.C. Sabourin, I. Salmon, S. Savic Prince, A. Scarpa, E. Schuuring, I. Serre, V. Siozopoulou, D. Sizaret, S. Smojver-Ježek, J. Solassol, K. Steinestel, J. Stojšić, C. Syrykh, S. Timofeev, G. Troncone, A. Uguen, S. Valmary-Degano, A. Vigier, M. Volante, S.G.F. Wahl, A. Stenzinger, M. Ilié


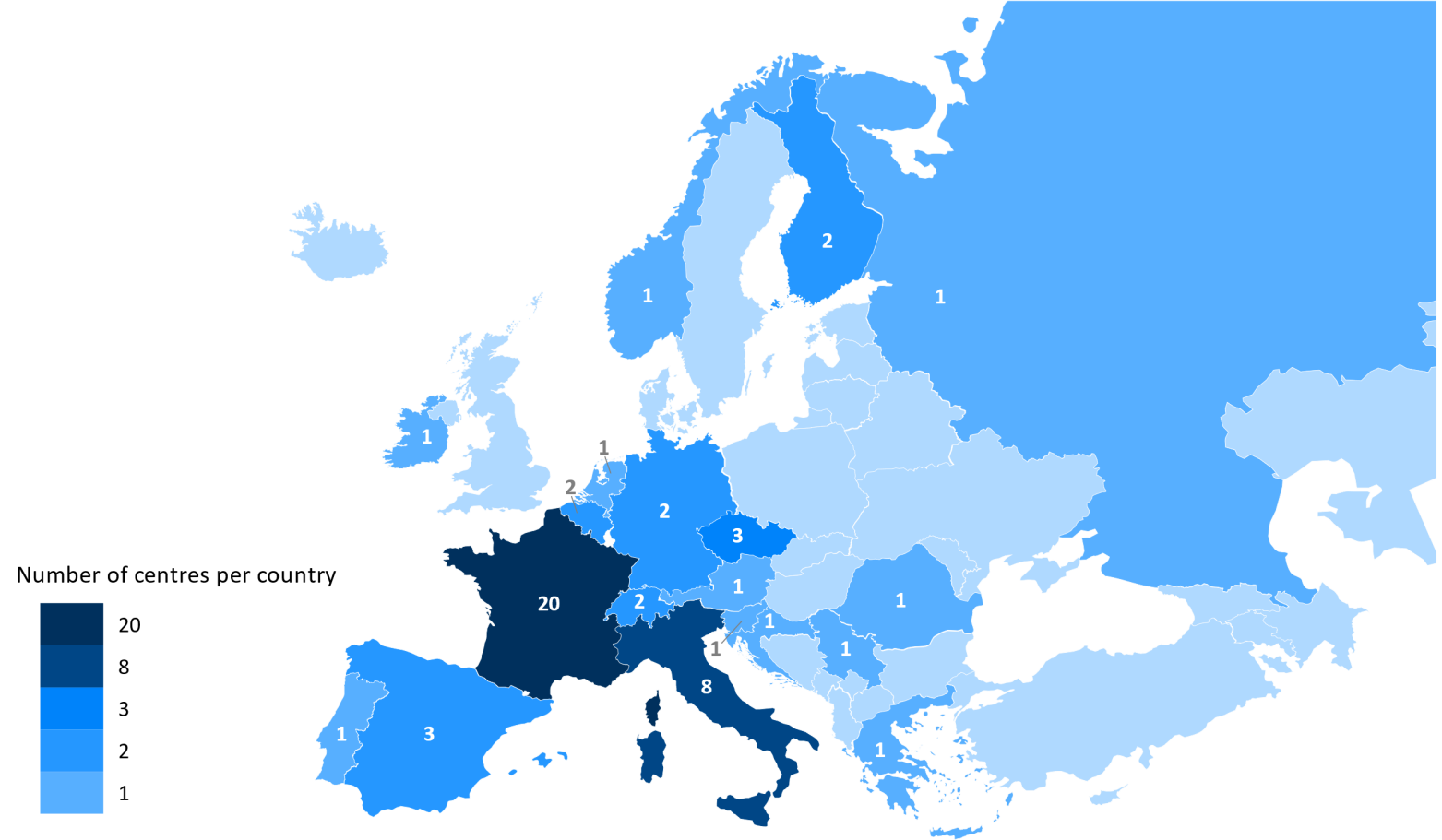


**Supplementary Figure S1. Number of laboratories in different countries across Europe that responded to the survey.**

In total, 19 European countries participated in the survey.

**Supplementary Figure S2. Laboratory accreditation across Europe (A) and by country (B–D). ISO, International Organization for Standardization; NGS, next-generation sequencing.**

**
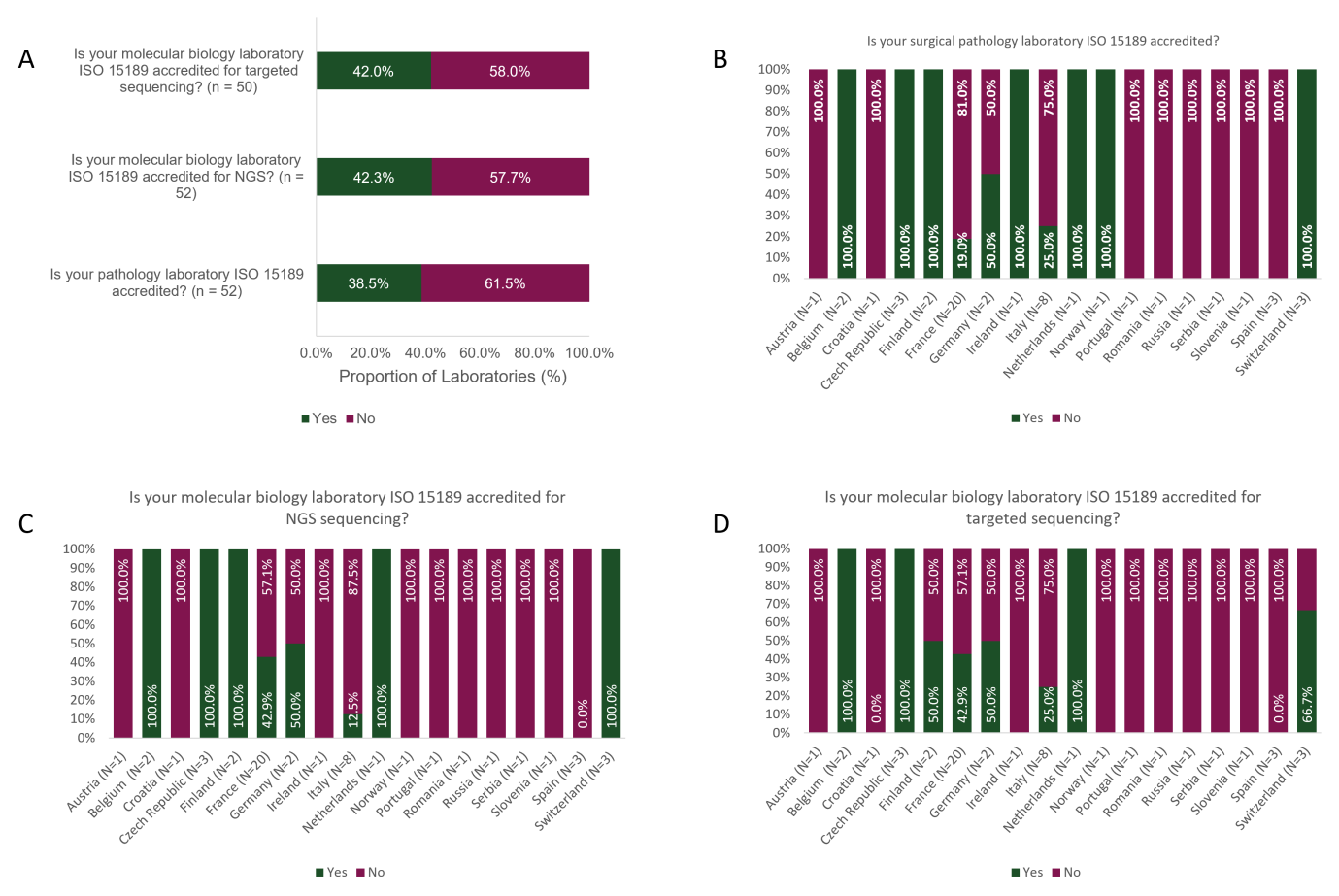
**

**Supplementary Table S1. Survey sent to pathology laboratories across Europe.**

| **Section 1 – About your laboratory**   1. Laboratory identification (please provide precise details): 2. Name (and telephone number) of the person completing the survey: 3. Name of the person in charge of the molecular biology division: 4. Name of the person in charge of the pathology division: |
| --- |
| **Section 2 – The circumstances in which you perform *EGFR* testing**   1. Do you perform *EGFR* testing only at the request of the clinician?    - Yes    - No 2. If you do not have a request from the clinician, do you routinely perform ‘reflex’ *EGFR* testing if you are aware of an advanced or metastatic tumour stage?    - Yes    - No 3. If you do not have a request from the clinician, do you routinely perform ‘reflex’ *EGFR* testing if the tumour stage is unknown?    - Yes    - No 4. If you do not have a request from the clinician, do you routinely perform ‘reflex’ *EGFR* testing in early stages (in particular, stages IB–IIIA)?    - Yes    - No   If yes, please indicate when you began testing in early stages (month/year, if known).   1. If you routinely perform ‘reflex’ *EGFR* testing in early stages (in particular, stages IB–IIIA), is this:    - Only on surgical specimens?    - Only on preoperative biopsies?    - Only on cytological specimens?    - On preoperative biopsies and surgical specimens?    - On preoperative biopsies, surgical specimens and cytological specimens?    - We don’t routinely perform ‘reflex’ *EGFR* testing in early stages    - Other (please specify)      1. Do you perform *EGFR* testing on cytological samples in advanced or metastatic stages?    - Yes, on cell blocks    - Yes, on smears    - Yes, on both cell blocks and smears    - No    - Other (please specify)      1. Do you routinely perform *EGFR* testing on liquid biopsies (blood) at diagnosis of advanced or metastatic stages?    - Yes, at diagnosis    - Yes, at tumour progression    - Yes, at both diagnosis and at tumour progression    - No 2. Do you routinely perform ‘reflex’ *EGFR* testing in the following cases of non-small cell lung carcinoma?  \|  \| All histological types \| Adenocarcinoma only \| NSCLC non-squamous only \| \| --- \| --- \| --- \| --- \| \| Yes \| 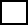 \| 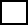 \| 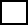 \| \| No \| 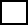 \| 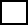 \| 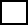 \| |
| **Section 3 – Molecular biology technique(s) performed**   1. Do you search for *EGFR* mutations using a targeted technique? If yes, by which technique on a **tissue or cytological** sample?    - No    - Yes, by Idylla™    - Yes, by cobas^®^    - Yes, by ddPCR    - Yes, by another technique (please specify) 2. Do you search for *EGFR* mutations using a targeted technique? If yes, by which technique on **blood** sample?    - No    - Yes, by Idylla™    - Yes, by cobas^®^    - Yes, by another technique (please specify) 3. Do you search for *EGFR* mutations using an NGS technique? If yes, by which technique?    - Yes, by hybrid capture    - Yes, by Amplicon    - No 4. Do you search for *EGFR* mutations using an NGS technique? If yes, on which samples?    - Yes, on tissue only    - Yes, on tissue and blood    - Yes, on tissue, blood and cytology    - No 5. According to the different techniques mentioned below. Please select all that apply.  \|  \| Yes \| No \| \| --- \| --- \| --- \| \| Do you only use targeted techniques in your laboratory? \| 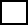 \| 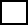 \| \| Do you only use NGS techniques in your laboratory? \| 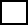 \| 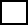 \| \| Do you use a targeted technique and then an NGS technique depending on the case? \| 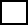 \| 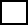 \| \| Do you systematically use a targeted technique then an NGS technique? \| 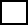 \| 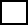 \| \| Do you systematically use an orthogonal technique if there is a discrepancy between the results of targeted sequencing and NGS sequencing? \| 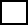 \| 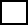 \| \| Do you use outsourced NGS tests on commercial platforms for *EGFR* mutations? \| 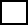 \| 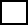 \| |
| **Section 4 – Percentage of tumour cells on tissue biopsies and indications**   1. What is your percentage tumour cell threshold for evaluation of *EGFR* status by a **targeted approach**?    - 10%    - 20%    - 30%    - Other (please specify) 2. What is your percentage tumour cell threshold for evaluation of *EGFR* status by an **NGS approach**?    - 10%    - 20%    - 30%    - Other (please specify) |
| **Section 5 – Turnaround time for *EGFR* status**   1. What do you mean by ‘time to results’?    - The time between the tissue (or blood) sample being taken by the physician, surgeon or nurse and the computer validation of the result    - The time between the registration of the sample in the pathology or biology laboratory and the computer validation of the result    - The time between the arrival of the sample in the molecular pathology/molecular biology sector and the computer validation of the result    - The time between obtaining the extracted and qualified nucleic acids and the computer validation of the result    - I do not know 2. What is the average time to obtain *EGFR* testing results in your circuit in the following scenarios? (Please fill in at least one field)  \| Between tissue sampling (biopsy) at your institution and computer validation of the result \|  \| \| --- \| --- \| \| Between the removal of the surgical specimen at your institution and computer validation of the result \|  \| \| Between the blood sample being taken at your institution and the computer validation of the result \|  \| \| Between the arrival of the sample in the cyto-pathology department and the computer validation of the result for an operative specimen (pulmonary lobectomy specimen without associated bone fragment) \|  \| \| Between the arrival of the sample in the cyto-pathology department and the computer validation of the result for a tissue biopsy \|  \| \| Between the arrival of the sample in the cyto-pathology department and the computer validation of the result for a liquid sample (BAL, pleural fluid, EBUS, etc.) \|  \| \| Between the arrival of the sample in the cyto-pathology department and the computer validation of the result for a cytological smear \|  \| \| Between the arrival of a blood sample (whole blood) and the computer validation of the result \|  \| \| Between the arrival of the sample (tissue curls) in the molecular pathology/molecular biology laboratory and the computer validation of the result \|  \| \| Between the arrival of the plasma at the molecular pathology/molecular biology laboratory and the computer validation of the result \|  \| \| Between the transmission of the extracted and qualified nucleic acids and the computer validation of the result \|  \| |
| **Section 6 – Annual rates of *EGFR* testing and testing results**   1. How many *EGFR* tests did you perform on tissue/cytology samples?   In 2018  In 2019  In 2020  In 2021   1. How many *EGFR* tests did you perform on liquid biopsies (blood)?   In 2018  In 2019  In 2020  In 2021   1. What is the percentage of *EGFR* mutation detected in your institution from tissue and cytological samples?  \|  \| 2018 \| 2019 \| 2020 \| 2021 \| \| --- \| --- \| --- \| --- \| --- \| \| Percentage total \|  \|  \|  \|  \| \| Percentage del19 and L858R \|  \|  \|  \|  \| \| Percentage exon 20 insertion \|  \|  \|  \|  \|  1. What is the percentage of *EGFR* mutation detected in your facility from liquid biopsy (blood)?  \|  \| 2018 \| 2019 \| 2020 \| 2021 \| \| --- \| --- \| --- \| --- \| --- \| \| Percentage total \|  \|  \|  \|  \| \| Percentage del19 and L858R \|  \|  \|  \|  \| \| Percentage exon 20 insertion \|  \|  \|  \|  \|  1. In what percentage of patients managed at your institution is *EGFR* testing performed?   In your institution?  Outside your institution? |
| **Section 7 – Accreditation**   1. Is your pathology laboratory ISO 15189 accredited?    - No    - Yes (please state when since) 2. Is your molecular biology laboratory ISO 15189 accredited for **NGS** sequencing?    - No    - Yes (please state when since) 3. Is your molecular biology laboratory ISO 15189 accredited for **targeted** sequencing?    - No    - Yes (Please state when since) |
| **Section 8 – External quality assessment**   1. Does your laboratory participate in external quality assessment (EQA) studies?    - Yes    - No   If yes, since when?   1. How often do you participate?    - Each year    - Every 2 years    - Not applicable (do not participate)    - Other (please specify)      1. What do you participate for?    - EQA for RT-PCR    - EQA for NGS    - Not applicable (do not participate) 2. Which EQA scheme(s) do you participate in? Please select all that apply.    - From the European Society of Pathology    - Gen&Tiss    - UK NEQAS    - EMQN    - Not applicable (do not participate)    - Other (please specify) |
| **Section 9 – Treatment**  Rather than *EGFR* testing, the questions in this final section concern the treatment of patients found to have an *EGFR* mutation. We are aware that information on treatment patterns may not be available to every respondent, but please answer the following questions if you can.   1. How many patients with an *EGFR* mutation received a targeted treatment with an EGFR inhibitor at your institution?   In 2018  In 2019  In 2020  In 2021   1. In which of the following settings was targeted therapy with an EGFR TKI offered to patients with an *EGFR* mutation?  \|  \| Second line \| First line \| Adjuvant \| None \| Don’t know \| \| --- \| --- \| --- \| --- \| --- \| --- \| \| 2018 \| 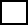 \| 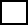 \| 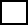 \| 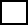 \| 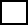 \| \| 2019 \| 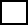 \| 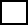 \| 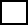 \| 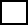 \| 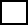 \| \| 2020 \| 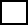 \| 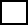 \| 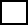 \| 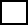 \| 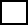 \| \| 2021 \| 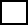 \| 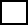 \| 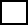 \| 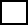 \| 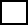 \|  1. Additionally, which generation(s) of EGFR TKI could patients receive?  \|  \| First Generation \| Second Generation \| Third Generation \| None \| Don’t know \| \| --- \| --- \| --- \| --- \| --- \| --- \| \| 2018 \| 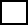 \| 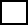 \| 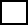 \| 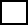 \| 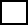 \| \| 2019 \| 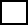 \| 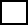 \| 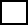 \| 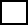 \| 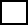 \| \| 2020 \| 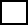 \| 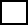 \| 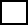 \| 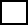 \| 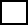 \| \| 2021 \| 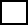 \| 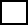 \| 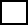 \| 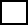 \| 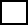 \| |

BAL, bronchoalveolar lavage; ddPCR, droplet digital polymerase chain reaction; EBUS, endobronchial ultrasound; *EGFR*, epidermal growth factor receptor gene; EMQN, European Molecular Genetics Quality Network; EQA, external quality assessment; ISO, International Organization for Standardization; NEQAS, National External Quality Assessment Scheme; NGS, next-generation sequencing; NSCLC, non-small cell lung cancer; RT-PCR, real-time polymerase chain reaction; TKI, tyrosine kinase inhibitor.

**Supplementary Table S2. Broad categories of targeted techniques.**

| - Allelic discrimination - SensiScreen^®^ FFPE assay - cobas^®^ - SNaPshot - EasyPGX^®^ Ready EGFR real-time PCR - Idylla™ EGFR Mutation Test - EasyPGX^®^ Ready EGFR - HRM - MassARRAY - Pyrosequencing (therascreen^®^ EGFR Pyro^®^ Kit, Qiagen) - qPCR - StripAssay^®^ ViennaLab, Real-Time PCR - Targeted quantitative PCR |
| --- |

EGFR, epidermal growth factor receptor; FFPE, formalin-fixed, paraffin-embedded; HRM, high-resolution melting; PCR, polymerase chain reaction; qPCR, quantitative polymerase chain reaction.

**Supplementary Table S3. EQA providers.**

| EQA provider | Number of participating laboratories |
| --- | --- |
| EMQN | 20 |
| Gen&Tiss | 19 |
| From the European Society of Pathology | 15 |
| UK NEQAS | 8 |
| QuiP | 4 |
| AFAQAP | 3 |
| Two different international ring trials | 1 |
| Dutch SKML | 1 |
| EQA LUNG | 1 |
| EuroClonality | 1 |
| GenQA | 1 |
| INSTAND | 1 |
| ISO 9001:2015 | 1 |
| National Survey of Pathology Scientific Society | 1 |
| SEKK (Czech EQA) | 1 |
| Spanish Society of Pathology | 1 |
| University of Hamburg | 1 |
| Not applicable (do not participate) | 4 |

AFAQAP, French Association for Quality Assurance in Pathological Anatomy and Cytology; EMQN, European Molecular Genetics Quality Network; EQA, external quality assessment; GenQA, Genomic Quality Assessment; ISO, International Organization for Standardization; NEQAS, National External Quality Assessment Scheme; QuiP, Quality in Pathology; SKML, Foundation for Quality Medical Laboratory Diagnostics.
